# Channelrhodopsin Excitation Contracts Brain Pericytes and Reduces Blood Flow in the Aging Mouse Brain *in vivo*

**DOI:** 10.3389/fnagi.2020.00108

**Published:** 2020-04-29

**Authors:** Amy R. Nelson, Meghana A. Sagare, Yaoming Wang, Kassandra Kisler, Zhen Zhao, Berislav V. Zlokovic

**Affiliations:** Department of Physiology and Neuroscience, Zilkha Neurogenetic Institute, University of Southern California, Los Angeles, CA, United States

**Keywords:** pericyte, optogenetics, channelrhodopsin, brain capillaries, red blood cell capillary flow

## Abstract

Brains depend on blood flow for the delivery of oxygen and nutrients essential for proper neuronal and synaptic functioning. French physiologist Rouget was the first to describe pericytes in 1873 as regularly arranged longitudinal amoeboid cells on capillaries that have a muscular coat, implying that these are contractile cells that regulate blood flow. Although there have been >30 publications from different groups, including our group, demonstrating that pericytes are contractile cells that can regulate hemodynamic responses in the brain, the role of pericytes in controlling cerebral blood flow (CBF) has not been confirmed by all studies. Moreover, recent studies using different optogenetic models to express light-sensitive channelrhodopsin-2 (ChR2) cation channels in pericytes were not conclusive; one, suggesting that pericytes expressing ChR2 do not contract after light stimulus, and the other, demonstrating contraction of pericytes expressing ChR2 after light stimulus. Since two-photon optogenetics provides a powerful tool to study mechanisms of blood flow regulation at the level of brain capillaries, we re-examined the contractility of brain pericytes *in vivo* using a new optogenetic model developed by crossing our new inducible pericyte-specific CreER mouse line with ChR2 mice. We induced expression of ChR2 in pericytes with tamoxifen, excited ChR2 by 488 nm light, and monitored pericyte contractility, brain capillary diameter changes, and red blood cell (RBC) velocity in aged mice by *in vivo* two-photon microscopy. Excitation of ChR2 resulted in pericyte contraction followed by constriction of the underlying capillary leading to approximately an 8% decrease (*p* = 0.006) in capillary diameter. ChR2 excitation in pericytes substantially reduced capillary RBC flow by 42% (*p* = 0.03) during the stimulation period compared to the velocity before stimulation. Our data suggests that pericytes contract *in vivo* and regulate capillary blood flow in the aging mouse brain. By extension, this might have implications for neurological disorders of the aging human brain associated with neurovascular dysfunction and pericyte loss such as stroke and Alzheimer’s disease.

## Introduction

Brains depend on blood flow for the delivery of oxygen and nutrients essential for proper neuronal and synaptic functioning (Zlokovic, 2011; Iadecola, 2017; Kisler et al., 2017a; Sweeney et al., 2019). Cerebral blood flow (CBF) is regulated by different cell types within the neurovascular unit (Zlokovic, 2011; Iadecola, 2017; Kisler et al., 2017a; Sweeney et al., 2019). The mammalian brain has developed a unique mechanism to control regional CBF known as neurovascular coupling. This mechanism ensures a rapid increase in the rate of blood flow and oxygen delivery to activated brain regions (Kisler et al., 2017a). Several studies have shown that smooth muscle cells (SMCs) on small brain arteries and arterioles, and pericytes on brain capillaries, physically contract and dilate small arteries and arterioles, and capillaries, respectively, to regulate blood flow responses to neuronal stimulation and/or neurotransmitters (reviewed in Attwell et al., 2016; Kisler et al., 2017a; Sweeney et al., 2018). However, the role of pericytes in regulating CBF has not been confirmed by all studies (Fernández-Klett et al., 2010; Hill et al., 2015; Wei et al., 2016).

French physiologist Rouget was first to describe pericytes in 1873 as regularly arranged longitudinal amoeboid cells on capillaries that have a muscular coat, implying that these are contractile cells that regulate blood flow (Rouget, 1873). There have been >30 publications from different labs, many of which from recent years, demonstrating that pericytes are contractile cells. This has been shown by *in vitro* studies using isolated brain, retinal and cochlear pericytes from different species (see Table 1 for details; Schor and Schor, 1986; Kelley et al., 1987, 1988; Das et al., 1988; Ferrari-Dileo et al., 1992; Haefliger et al., 1994, 1997, 2002; Murphy and Wagner, 1994; Chen and Anderson, 1997; Matsugi et al., 1997a,b,c; Dai et al., 2009, 2011; Neuhaus et al., 2017); *ex vivo* studies using cerebellar, cerebral and spinal cord slices and retinal microvessels or explants (see Table 2 for details; Schönfelder et al., 1998; Kawamura et al., 2003, 2004; Wu et al., 2003; Peppiatt et al., 2006; Yamanishi et al., 2006; Hall et al., 2014; Fernández-Klett and Priller, 2015; Mishra et al., 2016; Ivanova et al., 2017; Li et al., 2017; Zong et al., 2017; Alarcon-Martinez et al., 2019; Nortley et al., 2019); and *in vivo* studies in rodents (see Table 3 for details; Dai et al., 2009, 2011; Fernández-Klett et al., 2010; Hall et al., 2014; Hill et al., 2015; Biesecker et al., 2016; Mishra et al., 2016; Nelson et al., 2016; Wei et al., 2016; Bertlich et al., 2017; Kisler et al., 2017b; Hartmann et al., 2018; Khennouf et al., 2018; Alarcon-Martinez et al., 2019; Nortley et al., 2019). Recent optogenetic studies expressing light-sensitive channelrhodopsin-2 (ChR2) cation channels in mouse pericytes, however, were not conclusive. One using a chondroitin sulfate proteoglycan 4 (*Cspg4*) Cre-ChR2 mouse model did not show pericyte contractility after light stimulus (Hill et al., 2015), whereas the other one that used a platelet-derived growth factor receptor-β (*Pdgfrb*)-ChR2 mouse model demonstrated pericyte contractility (Hartmann et al., 2017, 2018). Another recent report suggested that capillaries do not dilate in response to neuronal stimulation, implying that pericytes do not control capillary diameter, although stimulation increased red blood cell (RBC) capillary flow (Wei et al., 2016).

**Table 1:** *In vitro* pericyte contractility.

Model	Preparation	Stimulus	Contraction	Reference
Bovine	Retina	Collagen gel matrix	Yes	Schor and Schor (1986)
Bovine	Retina	Cytochalasin B	Yes	Kelley et al. (1987)
Bovine	Retina	Adenosine triphosphate	Yes	Das et al. (1988)
Bovine	Retina	Histamine, serotonin, cyclic adenosine monophosphate	Yes	Kelley et al. (1988)
Bovine	Retina	Cholinergic and adrenergic agonists	Yes	Ferrari-Dileo et al. (1992)
Bovine	Retina	Sodium nitroprusside	Yes	Haefliger et al. (1994)
Rat	Retina	Angiotensin II and histamine	Yes	Murphy and Wagner (1994)
Bovine	Retina	Angiotensin II	Yes	Matsugi et al. (1997b)
Bovine	Retina	Partial pressure of carbon dioxide, angiotensin II	Yes	Matsugi et al. (1997c)
Bovine	Retina	Adenosine	Yes	Matsugi et al. (1997a)
Bovine	Retina	Sodium nitroprusside	Yes	Haefliger et al. (1997)
Bovine	Retina	Partial pressure of carbon dioxide	Yes	Chen and Anderson (1997)
Bovine	Retina	Sodium nitroprusside	Yes	Haefliger et al. (2002)
Guinea pig	Cochlea	Potassium, calcium, norepinephrine	Yes	Dai et al. (2009)
Guinea pig	Cochlea	Extracellular lactate	Yes	Dai et al. (2011)
Human	Brain	Vasoactive peptide endothelin-1	Yes	Neuhaus et al. (2017)

**Table 2:** *Ex vivo* pericyte contractility.

Model	Preparation	Stimulus	Contraction	Reference
Rat	Retina whole-mount	Angiotensin II, carbachol, bradykinin, histamine	Yes	Schönfelder et al. (1998)
Rat	Retina microvessels	Cholinergic agonists	Yes	Wu et al. (2003)
Rat	Retina microvessels	Adenosine triphosphate	Yes	Kawamura et al. (2003)
Rat	Retina microvessels	Angiotensin II	Yes	Kawamura et al. (2004)
Rat	Retina microvessels	Lactate	Yes	Yamanishi et al. (2006)
Rat	Retina whole-mount	Electrical stimulation or neurotransmitters	Yes	Peppiatt et al. (2006)
Rat	Cerebellar slices	Electrical stimulation or neurotransmitters	Yes	Hall et al. (2014)
Mouse	Brain slices	Thromboxane A2 agonist U46619	Yes	Fernández-Klett and Priller (2015)
Rat	Cortical slices	Thromboxane A2 agonist U46619	Yes	Mishra et al. (2016)
Rat	Retina whole-mount	Cannabinoid 2-arachidonoylglycerol and anandamide	Yes	Zong et al. (2017)
Rat	Spinal cord	Spinal cord injury	Yes	Li et al. (2017)
Mouse	Retina whole-mount	Electrical, P2Y-R agonist, nitric oxide donor, light	Yes	Ivanova et al. (2017)
Mouse	Retina whole-mount	Ischemia	Yes	Alarcon-Martinez et al. (2019)
Rat, Human	Cortical slices and surgically resected brain	Amyloid-beta_1–42_ oligomers	Yes	Nortley et al. (2019)

**Table 3:** *In vivo* pericyte contractility.

Model	Preparation	Stimulus	Contraction	Reference
Guinea pig	Cochlea	Potassium, calcium, norepinephrine	Yes	Dai et al. (2009)
Mouse	Cortex	Thromboxane A2 agonist U46619, cortical spreading depolarization	Yes	Fernández-Klett et al. (2010)
Guinea pig	Cochlea	Extracellular lactate	Yes	Dai et al. (2011)
Mouse	*in vivo*, cortex	Whisker stimulus	Yes	Hall et al. (2014)
Mouse	Cortex	Optogenic excitation of Channelrhodopsin-2	No	Hill et al. (2015)
Rat	Cortex	Forepaw stimulus	Yes	Mishra et al. (2016)
Mouse	Retina	Sensory stimulation	Yes	Biesecker et al. (2016)
Mouse	Cortex	Optogenic excitation of Channelrhodopsin-2	Yes	Hartmann et al. (2017, 2018)
Mouse	Cortex	Hind limb stimulus	No	Wei et al. (2016)
Guinea pig	Cochlea	Tumor necrosis factor	Yes	Bertlich et al. (2017)
Mouse	Somatosensory cortex	Hind limb stimulus	Yes	Kisler et al. (2017b)
Mouse	Cortex	Whisker pad stimulus or cortical spreading depolarization	Yes	Khennouf et al. (2018)
Mouse	Cortex	Amyloid-beta peptides	Yes	Nortley et al. (2019)
Mouse	Retina	Ischemia	Yes	Alarcon-Martinez et al. (2019)

Since two-photon optogenetics provides a powerful tool for studying the mechanisms of blood flow regulation at the level of brain capillaries, we re-examined the contractility of brain pericytes *in vivo* using a new optogenetic model developed by crossing our new inducible pericyte-specific CreER mouse line (Nikolakopoulou et al., 2019) with ChR2 mice (Madisen et al., 2012). We induced the expression of ChR2 in pericytes by tamoxifen, activated ChR2 by 488 nm excitation light, and monitored pericyte contractility, brain capillary diameter changes, and RBC flow velocity in aged mice by *in vivo* two-photon microscopy. Since many studies have shown that a rise in intracellular calcium causes pericytes to contract (Wu et al., 2003; Kawamura et al., 2004; Peppiatt et al., 2006; Yamanishi et al., 2006; Dai et al., 2009; Khennouf et al., 2018; Alarcon-Martinez et al., 2019), we hypothesized that light-induced excitation of ChR2 in pericytes *in vivo* will depolarize pericytes causing them to contract and constrict the underlying capillary, which in turn will reduce the capillary flow of RBCs.

## Materials and Methods

### Mice

**Figure 1:**
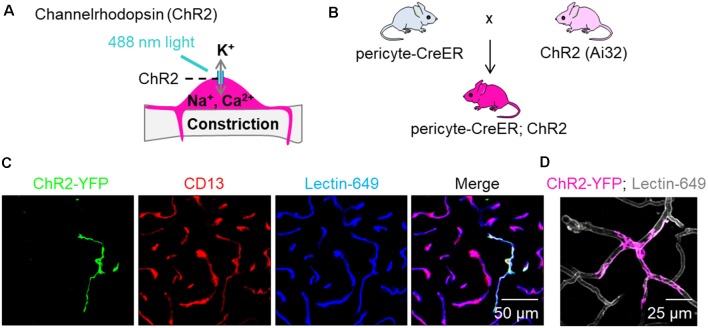
Expression of channelrhodopsin (ChR2) in pericytes. **(A)** Schematic demonstrating our hypothesis that excitation of ChR2 expressing pericytes by 488 nm light will depolarize pericytes causing them to contract and constrict the underlying capillary. **(B)** Schematic showing the breeding scheme of recently characterized double promoter pericyte-CreER mice with tamoxifen inducible Cre-recombinase expression in pericytes, with mice that have a loxP-flanked STOP cassette that is excised in the presence of Cre to drive ChR2-EYFP fusion protein expression in pericytes (Ai32, Jackson Laboratory); the crossed mice were termed pericyte-CreER; ChR2. **(C)** Representative images of EYFP expression (green) showing co-localization of ChR2 cation channels with CD13+ pericytes (red) on Lectin-649+ capillary profiles (blue) in pericyte-CreER; ChR2 mice treated with tamoxifen (40 mg/kg i.p. daily for 4 days) and studied 2 weeks after the last tamoxifen injection. **(D)** *In vivo* z-stack maximum projection image demonstrating EYFP (pink) in a pericyte along lectin-649 + (gray) capillary profiles in a 32-month-old female pericyte-CreER; ChR2 mouse.

We utilized a recently developed and characterized pericyte-specific CreER mouse line generated by a double-promoter strategy using a combination of *Pdgfrb* and *Cspg4* promoters to drive CreER expression in pericytes (Nikolakopoulou et al., 2019). Briefly, *Pdgfrb* and *Cspg4* transgenic constructs were generated, one expressing Flippase recombinase (Flp) under the control of the *Pdgfrb* promoter, and the other carrying an Frt-Stop-Frt-CreER cassette (Frt: flippase recognition target; CreER: recombinant protein between Cre recombinase and a mutated ligand binding domain of the estrogen receptor) under the control of the *Cspg4* promoter (Nikolakopoulou et al., 2019). To test pericyte contractility, we utilized ChR2, a non-selective cation channel permeable to sodium, potassium and calcium that opens upon stimulation with 488 nm light and depolarizes the cell (Figure 1A). ChRs were initially used as tools to depolarize neuronal membranes (Zhang et al., 2006, 2007), but have also been used to study contractility of non-neuronal cells such as SMCs (Hill et al., 2015), cardiac myocytes (Johnston et al., 2017), and brain pericytes (Hill et al., 2015; Hartmann et al., 2017, 2018). We crossed pericyte-CreER mice (Nikolakopoulou et al., 2019) with mice that have a loxP-flanked STOP cassette that is excised in the presence of Cre to drive ChR2-EYFP fusion protein expression (Ai32, The Jackson Laboratory; Figure 1B). The progeny mice were termed pericyte-CreER; ChR2. Upon four consecutive daily injections of tamoxifen (40 mg/kg i.p.), approximately 40% of pericytes expressed Cre, as previously reported (Nikolakopoulou et al., 2019). ChR2 was expressed primarily in pericytes as shown by co-localization of ChR2-EYFP fusion protein with CD13+ pericytes on brain tissue sections 2 weeks after the last tamoxifen injection (Figure 1C), which was also visualized *in vivo* (Figure 1D; Supplementary Video S1).

**Figure 2:**
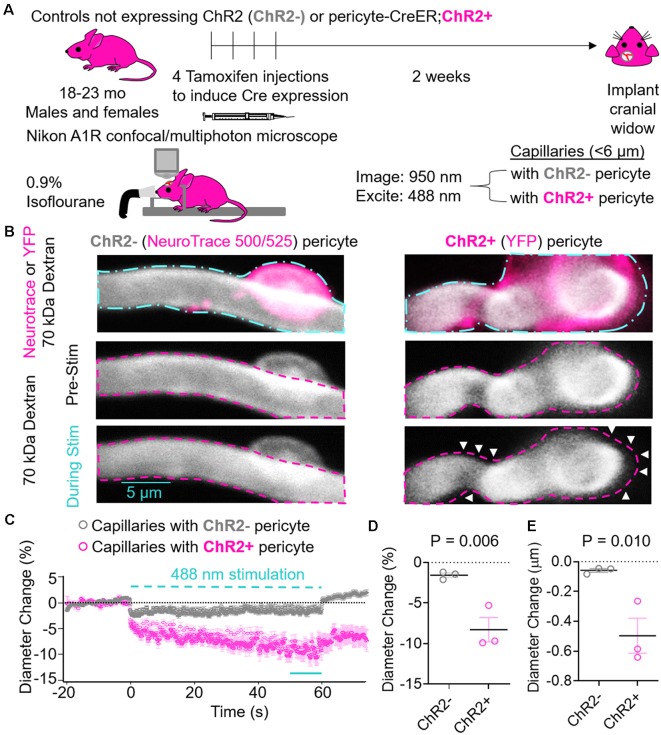
Effects of ChR2 excitation in pericytes on the underlying capillary diameter. **(A)** Schematic of experimental paradigm. Control mice not expressing ChR2 (ChR2−; 18-month old, two males, one female, *n* = 3) or Pericyte-CreER; ChR2 (ChR2+; 23-month old, two males and one female, *n* = 3) mice received four consecutive tamoxifen injections (40 mg/kg i.p. daily), and 2 weeks later cranial windows were implanted and imaging was performed on a Nikon A1R confocal/multiphoton microscope under light anesthesia using 0.9% isofluorane. We used 950 nm light for imaging and 488 nm light for excitation of ChR2 cation channels. Capillaries (<6 μm) with ChR2− or ChR2+ pericytes were imaged before, during, and after light stimulus. **(B)** Representative images of capillaries with ChR2− pericytes visualized by NeuroTrace 500/525 (left) and ChR2+ pericytes visualized by YFP expression (right). The cyan dashed line (top panels) indicates regions of interest stimulated with 488 nm light, and the fuschia dashed lines indicate the capillary diameters before stimulation. White arrows indicate areas of capillary constriction. **(C)** Average time courses by mouse of capillary diameter changes of capillaries covered with ChR2− pericytes (gray circles) or ChR2+ pericytes (fuschia circles). Each single point along the time axis represents mean ± SEM, from *n* = 25 capillaries with ChR2+ pericytes from three pericyte-CreER; ChR2 mice and *n* = 29 capillaries with ChR2− pericytes from three control mice not expressing ChR2. Capillary diameter before stimulation was arbitrarily taken as zero (0). The dashed cyan line indicates the 60 s 488 nm stimulation period. The unbroken cyan line indicates the 10 s period quantified in panels **(D,E)**. **(D)** Quantification of relative mean capillary diameter change (%) by mouse for ChR2− (gray circles) vs. ChR2+ pericyte (fuschia circles) capillaries over the last 10 s period during stimulation indicated in **(C)**. **(E)** Quantification of absolute mean capillary diameter change (μm) by mouse for ChR2− (gray circles) vs. ChR2+ (fuschia circles) pericyte capillaries over the last 10 s period during stimulation indicated in **(C)**. In **(D,E)** mean ± SEM, individual values averaged per mouse derived from *n* = 25–29 pericyte-covered capillaries from three mice per group. *P* = 0.006 and 0.010, respectively by one-tailed student’s *t*-test.

**Figure 3:**
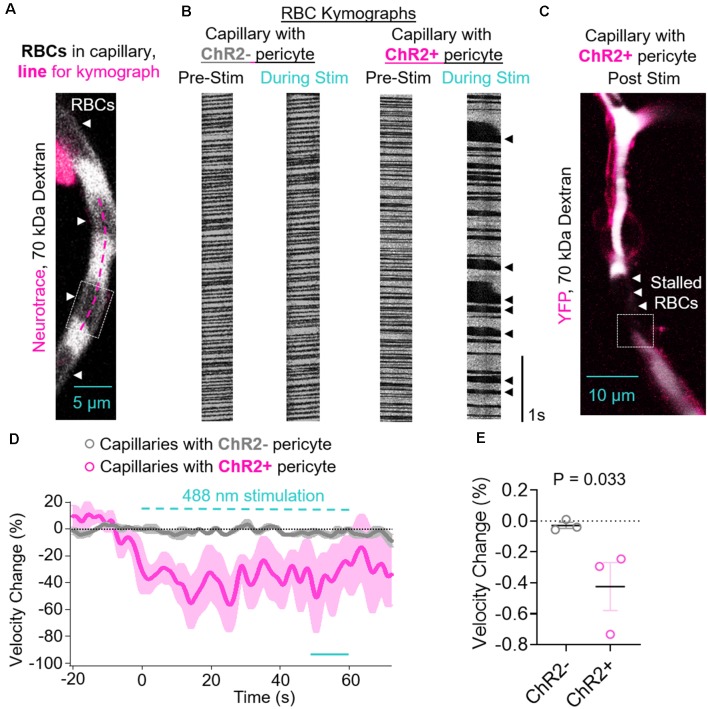
Contraction of ChR2-positive pericytes leads to reduced capillary red blood cell (RBC) velocity. **(A)** Representative image of a capillary with a ChR2− pericyte demonstrating how RBCs are visualized in locations that are not 70 kDa dextran-positive, and an example line for a kymograph drawn along the length of the capillary (fuschia dashed line). The dashed box indicates the location shown in Supplementary Video S2. **(B)** Representative RBC kymographs acquired pre-stimulation and during light stimulation (488 nm) of capillaries with a ChR2− or ChR2+ pericyte. Black arrows indicate areas of slowed or stalled RBC flow. **(C)** Representative lower magnification image of the vessel segment with ChR2+ pericyte visualized in panel **(B)** showing stalled RBC in brain capillaries after stimulation of ChR2+ pericytes. The dashed box indicates the location shown in Supplementary Video S3. **(D)** Average time courses by mouse of capillary RBC velocity changes for capillaries covered with ChR2− pericytes (gray circles) or ChR2+ pericytes (fuschia circles). Each single point along the time axis represents the mean ± SEM, from *n* = 26 capillaries with ChR2− pericytes from three control mice that do not express ChR2 and *n* = 14 capillaries with ChR2+ pericytes from three pericyte-CreER; ChR2 mice. RBC velocity before stimulation was arbitrarily taken as zero (0). The dashed cyan line indicates the 60 s 488 nm stimulation period. The unbroken cyan line indicates the 10 s period during stimulation quantified in panel **(E)**. **(E)** Quantification of mean reduction in capillary RBC velocity (%) by mouse for ChR2− (gray circles) vs. ChR2+ pericyte (fuschia circles) capillaries over the last 10 s period during stimulation indicated in **(D)**. Mean ± SEM, individual values averaged per mouse derived from *n* = 14–26 pericyte-covered capillaries from three mice per group. *P* = 0.033, by one-tailed student’s *t*-test.

Since neurovascular dysfunction is typically observed in neurological disorders associated with aging, such as stroke and neurodegenerative diseases including Alzheimer’s disease (Sweeney et al., 2018, 2019), we used 23–32-month-old mice in these studies. We also used 18 month old control mice that do not express ChR2. A 32-month-old mouse was only used to visualize ChR2YFP+ pericytes on lectin+ capillaries (Figure 1D and Supplementary Video S1) and was not used in any statistical analysis. All statistical analysis in pericyte-CreER; ChR2 mice in Figures 2, 3 was performed in 23-month-old mice. Both male and female animals were used for experiments. Mice were housed in plastic cages on a 12 h light cycle with *ad libitum* access to water and a standard laboratory diet. During *in vivo* surgery and experiments, body temperature was maintained with electric heating pads, with thermal feedback and respiration monitoring. Intraperitoneal injections of 5% glucose in isotonic saline (0.2 ml per 25 g) were administered every 2 h. Experiments were performed under isoflurane anesthesia (SomnoSuite, Kent Scientific), unless otherwise specified. For details, see specific experiments below.

### Cranial Window Implantation

Mice were initially anesthetized with 100 mg/kg of ketamine and 50 mg/kg of xylazine and fixed in a stereotaxic frame (Kopf Instruments). A circular cranial window was drilled over the hindlimb region of the somatosensory cortex (center at AP = −0.94 mm, *L* = 1.5 mm) and the underlying dura was removed. To visualize pericytes in control mice that did not express ChR2 or YFP, 50–100 μl of NeuroTrace 500/525 dye [1:25 dilution in sterile artificial cerebrospinal fluid (aCSF)] was applied topically to the exposed cortical surface of the open cranial window for 5 min, then thoroughly washed with sterile aCSF (Damisah et al., 2017). The window was filled with sterile aCSF and covered with a 3-mm round coverslip. Mice were transitioned to isoflurane following cranial window implantation.

### *In vivo* Multiphoton and Confocal Microscopy

In these studies, we used a Nikon A1R multiphoton and confocal microscopy system with NIS-Elements software control (25× objective, 1.1 numerical aperture, point spread function is 0.300 μm). For *in vivo* multiphoton imaging mice were anesthetized with 0.9% isoflurane. The vasculature was labeled *via* retro-orbital injection of 75 μl of 70 kDa Texas Red-dextran (0.1 ml of 10 mg/ml) or DyLight 649 labeled Lycopersicon Esculentum (Tomato) Lectin (1 mg/ml, DL-1178, Vector Labs) and imaged through the cranial window. In control mice not expressing ChR2, we utilized a previously described fluoro-Nissl dye that labels capillary pericyte somas that are alpha-smooth muscle cell actin (α-SMA)-negative (Damisah et al., 2017) to visualize and stimulate capillaries with a pericyte, including the soma. *In vivo* images were acquired at depths up to 200 μm below the pial surface using a mode-locked Ti:sapphire laser (Insight DS+; Spectra Physics) set to 950 nm (power ~10 mW) for EYFP and Texas Red excitation. The image acquisition area (512 × 64 pixels) was restrained to maintain high spatial resolution (0.12 μm/pixel) and a fast frame rate (118.2 frames per second) to accurately measure RBC flow changes. Glucose in isotonic saline 5% weight/volume solution (200 μl/25 g mouse) was applied every 2 h i.p. for the duration of experiment, as we previously reported (Kisler et al., 2018).

### Pericyte Stimulation

A region of interest was identified and set as the stimulation area by the NIS-Elements software to outline the pericytes on the capillary segment in the visible field. Concurrently with multiphoton imaging, 488 nm light excitation from the confocal microscope laser source (power ~2.5 mW) was triggered using a set protocol which consisted of a 20 s baseline (no 488 nm illumination), followed by 40 s of 488 nm stimulation (0.1 s duration, 1 s intervals). We did not collect topological information about where the stimulated capillaries were located, but since our pericyte-CreER line and the NeuroTrace dye labeled SMA-negative pericytes on capillaries (<6 μm), the stimulated cells were likely mid-capillary pericytes.

### Capillary Diameter Measurements

For diameter measurements, images of the 70 kDa Texas Red-dextran signal were taken using the resonance scanner at 60–120 frames per second, generating an image of the vessel over time. Using NIS-Element-Advanced Research software, 3–5 lines were drawn perpendicular to the capillary equidistant along the length of the vessel approximately 5–10 μm apart to generate a kymograph, where the vessel diameter was recorded as a line of pixels in one axis, and time in the other, as we previously reported (Kisler et al., 2018). We did not measure diameter directly under the soma of pericytes in either ChR2+ or ChR2− mouse capillaries due to NeuroTrace dye fluorescence bleed through of the pericyte soma signal in ChR2− mice. Each kymograph was thresholded and analyzed with custom protocols written in Igor Pro 6 (WaveMetrics), as we previously reported (Kisler et al., 2018): first, images were smoothed with a 3 × 3 pixel Gaussian filter to remove noise and thresholded using the Igor Pro built-in “fuzzy entropy” threshold routine to generate a black-and-white (binary) image of the vessel diameter vs. time. The Igor Pro custom analysis protocol identified the transitions between black/white and white/black in each line of the image, indicating the edges of the vessel, and then calculated the width of the vessel for each line in the image. The resulting diameter data were low-pass filtered (1 Hz cut off) and notch filtered (0.5 Hz), then smoothed with a 1 s window box filter. Vessels with basal diameter <6 μm were considered capillaries. Basal diameters were calculated as an average over 20 s prior to stimulus, with basal diameters set to zero. For each capillary, 3–5 lines were averaged together to represent an individual capillary diameter. Diameter changes during stimulus were determined as a percent of basal diameter. For statistical comparisons, the percentage average diameter changes (Figure 2D) and absolute mean diameter changes (Figure 2E) over the last 10 s of stimulation (50–60 s after stimulation start) of individual values averaged per mouse from ChR2+ pericyte capillaries were calculated and statistically compared to ChR2− pericytes from *n* = 25–29 pericyte-covered capillaries from three mice per group.

### Red Blood Cell (RBC) Velocity Measurements

For RBC velocity, only 70-kDa Texas Red-dextran was used for imaging. Kymographs were generated parallel to each capillary along the vessel midline. RBCs appear as dark spots in the dye-labeled blood plasma. Velocity data were analyzed using a MatLab algorithm as previously described (Kim et al., 2012; Kisler et al., 2018) and then low-pass filtered (1 Hz cut off) and notch filtered (0.5 Hz), then box filtered with a 1 s window to remove heartbeat and breathing artifacts using custom written protocols in Igor Pro to automate the filtering process, as we previously described (Kisler et al., 2018). Basal RBC velocities were calculated as an average over 20 s prior to stimulus, with individual basal capillary RBC velocities set to zero. RBC velocity changes during stimulus were determined as a percentage of basal velocity. For comparison in Figure 3E, individual values were averaged per mouse from ChR2+ and ChR2− pericyte capillary RBC velocities over the last 10 s of stimulation (averaging between 50 s and 60 s after stimulation start) were used to determine the average velocity change during stimulus and statistically compared.

### Immunohistochemistry of ChR2-EYFP Protein Co-localization With Pericytes

Mice were anesthetized with 4% isoflurane and then transcardially perfused with 50 ml of phosphate buffered saline containing ethylenediaminetetraacetic acid. Brains were removed and embedded into O.C.T. compound (Tissue-Tek) on dry ice. They were cryosectioned at a thickness of 18 μm. The sections were cut sagittally and those from depths of 50–150 μm, to coincide with TPLSM imaging depths, were used for assessment. The sections were subsequently blocked with 5% normal donkey serum (Vector Laboratories) and Triton (0.05%) for 1 h and incubated in goat anti-CD13 (1:200) primary antibody (Kisler et al., 2017b) diluted in donkey serum blocking solution overnight at 4°C. To visualize CD13+ pericytes, donkey anti-goat IgG, Alexa 568 (1:500; Invitrogen, A-11057) secondary antibody was used for incubation for 1.5 h at room temperature. To visualize brain microvessels, sections were incubated with fluorescein-conjugated L. esculentum lectin (Vector Laboratories FL-1171, 1:200). In each animal five randomly selected fields from the cortex were analyzed in six nonadjacent sections (~100 μm apart).

### Statistics

Since ChR2-positive and ChR2-negative capillaries were assessed within the same animals, experiments were analyzed, but not performed, in a blinded fashion. Sample sizes were calculated using nQUERY assuming a two-sided α-level of 0.05, 80% power, and homogenous variances for the two samples to be compared, with the means and common s.d. for different parameters predicted from published data (Peppiatt et al., 2006; Hall et al., 2014; Mishra et al., 2016; Nortley et al., 2019) and our previous studies (Kisler et al., 2017b, 2018). Using nQUERY, we calculated that a sample size of three mice in each group will have 80% power to detect a difference in means of 6% diameter change (the difference between a Group 1 mean of 8% and a Group 2 mean of 2%) assuming that the common standard deviation is 2 using a two group *t*-test with a 5% one-sided significance level. Thus, we used three mice per group. Individual values in each mouse are averaged from *n* = 14–29 pericyte-covered capillaries. All data are expressed as mean ± SEM. Normality was determined with a Shapiro–Wilk test, and statistical significance was determined by a one-tailed student’s *t*-test (GraphPad Prism 8.1). The accepted level of statistical significance was *P* ≤ 0.05.

## Results

### Excitation of ChR2-Positive Pericytes Contracts the Cell and Constricts the Underlying Capillary

Pericyte-CreER; ChR2 mice at 23 months of age and control mice not expressing ChR2 at 18 months of age were administered daily tamoxifen (40 mg/kg i.p.) for four consecutive days. Approximately 2 weeks later, cranial windows were implanted over the hindlimb somatosensory cortex. Mice were fixed in a stereotaxic frame under 0.9% isoflourane anesthesia and imaged with 950 nm light. Capillaries defined as vessels <6 μm in diameter with ChR2− or ChR2+ pericytes were imaged and stimulated on a Nikon A1R confocal and multiphoton microscope, and the diameter of the underlying capillary was measured (Figure 2A). Intermittent 60 s stimulation with 488 nm light caused ChR2+ pericytes to contract and constrict the underlying capillary resulting in a significant reduction in capillary diameter compared to capillaries with ChR2− pericytes (Figures 2B,C, Supplementary Videos S2, S3). The kinetics of the constriction during light stimulation had a rapid phase within the first few seconds in both capillaries with ChR2− and ChR2+ pericytes, though to a much greater degree in capillaries with ChR2+ pericytes. There was also a slower phase of contraction over tens of seconds occurring in capillaries with ChR2+ pericytes (Figure 2C). Previous studies have shown light-induced stimulation and contraction of neurovascular SMCs resulted in constriction of the underlying arteriole (Choi et al., 2010; Kimbrough et al., 2015). Therefore, it is possible that pericytes, or perhaps endothelial cells, have similar light-induced capabilities causing contraction possibly explaining why we observed a small diameter decrease in capillaries with ChR2− pericytes. Excitation of ChR2+ pericytes resulted in an 8.29 ± 1.51% and 0.50 ± 0.01 μm decrease in capillary diameter compared to baseline values, whereas ChR2− pericytes stimulated in a similar way showed only a small decrease in capillary diameter of 1.56 ± 0.47% and 0.06 ± 0.02 μm (Figures 2D,E; mean ± SEM; *p* = 0.006 and 0.010, respectively).

### Pericyte Contraction Leads to Reduced RBC Velocity

Since, we found a significant decrease after light stimulation in the capillary diameter in capillaries covered with ChR2+ pericytes, we next studied if RBC flow was affected after pericyte stimulation. The contraction of ChR2+ pericytes upon 488 nm stimulation and constriction of the underlying capillary led to a reduction in RBC velocity compared to the velocity before stimulation (Figures 3A–D). This was visualized by irregular RBCs kymographs (Figure 3B) and stalled RBCs appeared as black gaps in the white pseudo colored 70 kDa Texas red-dextran in the capillary after ChR2 excitation (Figure 3C). RBC velocity was decreased during light stimulation in capillaries with ChR2+ pericytes, with minimal changes observed in ChR2− capillaries, averaged by mouse (Figure 3D). Compared to ChR2− pericytes, there is greater variability in RBC velocity change during stimulation of ChR2+ pericyte capillaries as in some capillaries the RBC flow not only slows down but also stalls and occasionally speeds up, possibly due to backlogged pressure in the stalled vessels (Figure 3D). Upon excitation of ChR2+ pericytes, RBC capillary velocity was reduced resulting in an average reduction of 42.44 ± 15.55% compared to the respective RBC velocity values before excitation, and significantly reduced compared to the excitation of ChR2− pericytes (Figure 3E, mean + SEM; *p* = 0.03).

## Discussion

Using two-photon optogenetics and our new pericyte-CreER; ChR2 mouse line (Figure 1), here we show that pericytes contract brain capillary diameter and regulate capillary blood flow in the aging mouse brain. Our findings are consistent with the majority of previous studies suggesting that pericytes are contractile cells that control CBF, including studies using isolated brain, retinal and cochlear pericytes (see fully referenced Table 1), *ex vivo* cerebellar, cerebral and spinal cord slices, and retinal microvessels or explants (see fully referenced Table 2), and *in vivo* studies in rodents (see fully referenced Table 3). The present study shows that light excitation of the ChR2 cation channel in pericytes directly leads to their contraction on brain capillaries <6 μm in diameter, which in turn leads to constriction of the underlying capillary and a significant 8% decrease in capillary diameter (Figure 2) accompanied by a 42% reduction in RBC flow during stimulation including some capillaries with stalled RBCs flow (Figure 3).

That pericytes are contractile cells is supported by single-cell RNAseq studies showing that mouse pericytes express several genes encoding for contractile proteins such as vimentin, desmin, titin, myosin heavy chains 9, 10 and 11, and myosin light chains 6, 9, 12a, and 12b, and other myosin genes (Myo1c, 1d, 5a, 6, 10 and 18a; He et al., 2018; Vanlandewijck et al., 2018). It has been debated whether or not pericytes can express low levels of α-SMA as this is typically considered as an SMC contractile protein (Hill et al., 2015; He et al., 2018; Vanlandewijck et al., 2018). Interestingly, it has been shown that the detection of α-SMA in capillary pericytes by immunostaining requires prevention of filamentous actin depolymerization, which when controlled, shows detectable levels of α-SMA protein in pericytes (Alarcon-Martinez et al., 2018). Nonetheless, a recent study suggested that optogenetic stimulation of pericytes lacking α-SMA produces a decrease in capillary diameter in the living mouse brain (Hartmann et al., 2018), supporting the concept that contractile proteins other than α-SMA can mediate pericyte contraction. Furthermore, our results provide evidence that α-SMA-negative pericytes likely use some alternative contractile mechanism to alter capillary tone. Altogether, these studies suggest that pericytes express contractile protein machinery that is different from SMCs. This, again, is consistent with their role in regulating blood flow in capillary vessels with a much narrower diameter compared to arterioles and small arteries, which might require a more robust contractile protein system. The exact role of proteins that mediate pericyte contraction remains, however, unclear at present. This could possibly be determined by future studies using our new pericyte-CreER model to delete specific pericyte genes encoding different contractile proteins from the brain.

A recent optogenetic study using *Cspg4*-driven ChR2 expression found that SMCs, but not pericytes, contract upon ChR2 stimulation (Hill et al., 2015). Although it is not entirely clear why this earlier study (Hill et al., 2015) was not able to show that pericytes contract after ChR2 excitation, there are several possibilities that could potentially account for the differences between the previous and the present study. It has been found that SMCs and pericytes express different contractile proteins and calcium channels (Kisler et al., 2017a; Vanlandewijck et al., 2018). This raises a possibility that a threshold for the optogenetic stimulation of ChR2 between SMCs and pericytes linked to contractility could also be different and might depend on the source and duration of the light stimulus. With regards to the potential differences in sensitivity to ChR2 stimulation between SMCs and pericytes, future studies should investigate the threshold of ChR2 stimulation between these two cell types with concurrently expressing genetically encoded calcium indicators, such as GCaMP6, to measure how much intracellular calcium rise is required to induce contractility in each of these two cell types vs. the duration and source of light stimulus.

Furthermore, we used 0.9% isoflurane, the minimum dose required to lightly anesthetize mice during our experiments to reduce potential off-target effects of anesthesia. Previous studies have shown that inhaled anesthetics have neurovascular effects. For example, 1–3% sevoflurane, but not 1–3% isoflurane, increases BBB breakdown in aged rats (Acharya et al., 2015). Furthermore, 2% isoflurane has vasomotor effects that either constricts or dilates vessels depending on the tone of vessels at the time of administration (Park et al., 1998). Hill et al. (2015) used ketamine/xylazine anesthetic during ChR2 imaging experiments, which increases blood flow by vasodilation (Oren et al., 1987) and alters neuronal function impairing neurovascular coupling (Masamoto and Kanno, 2012). Future studies should evaluate the impact of ChR2 excitation of pericytes in awake mice.

Other factors such as the resolution of the microscope may also play a role in detecting pericyte contractility and diameter changes since the resting diameter of arteries and arterioles is quite large, and the contractility of SMCs and alteration of the underlying vessel diameter is much larger and easier to detect compared to the resting diameter of capillaries. Thus, observing significant changes in capillary diameter requires higher resolution and carefully considered experimental and statistical design, as previously discussed (Kisler et al., 2018). Recent *in vivo* studies reported that hindlimb or forepaw stimulation for 10 s (Mishra et al., 2016; Kisler et al., 2017b) and whisker pad stimulation for 15 s (Hall et al., 2014) both lead to capillary dilation ahead of arterioles, which resulted from isotonic pericyte relaxation and correlated with the degree of pericyte coverage. On the other hand, a reduced time of whisker pad stimulation of 2 s was not sufficient to produce changes in capillary diameter despite producing an increase in RBCs capillary flow (Wei et al., 2016); this could possibly result from an initial isometric relaxation of pericytes that with longer stimulation as used in other studies (Hall et al., 2014; Mishra et al., 2016; Kisler et al., 2017b) converts into isotonic relaxation causing a detectable change in capillary diameter. Because the present study was focused on constrictive responses of pericytes, it would be difficult to make any direct comparison with previous studies focused on dilation of capillaries and relaxation of pericyte tone. However, future optogenetic studies using hyperpolarization models of pericytes expressing, for example, halorhodopsin (e.q. eNpHR3.0) or archaerhodopsin-3 (e.q. ArchT) channels (Madisen et al., 2012), should be able to directly address the relationship between pericyte relaxation and dilation of the underlying capillary.

Recent studies found that Alzheimer’s amyloid-β (Aβ) oligomers induced pericytes to contract and constrict the underlying capillary in acute cerebral slices from rats and humans, and that capillaries covered with pericytes were typically constricted in Alzheimer’s disease brains post-mortem (Nortley et al., 2019). Since pericyte contractility may provide the force for intramural periarterial drainage at the capillary level (Aldea et al., 2019) this could potentially impact periarterial drainage of Aβ (Diem et al., 2016) and/or its trans-vascular clearance across the BBB (Shibata et al., 2000; Deane et al., 2004; Nelson et al., 2017), or *via* meningeal lymphatic vessels (Da Mesquita et al., 2018). Future studies should therefore investigate how Aβ directly interacts with a receptor on brain pericytes to trigger contractility, and how this could influence perivascular flow, BBB and meningeal lymphatic clearance, and BBB integrity, given that early pericyte injury is shown to be an early independent biomarker of human cognitive dysfunction (Montagne et al., 2015; Nation et al., 2019).

Altogether, our findings show that pericytes contract *in vivo* and regulate capillary blood flow in the aging mouse brain which might have broader implications for the understanding of CBF regulation in the aging human brain, particularly in neurological diseases associated with pericyte loss, degeneration and neurovascular dysfunction. This includes disorders such as stroke, Alzheimer’s disease and possibly other neurodegenerative diseases (Sweeney et al., 2018, 2019). Future studies should compare young vs. old mice to determine if there is an effect of aging on pericyte contractility. Furthermore, prospective studies should also provide a better understanding of how age-dependent pericyte dysfunction occurs in different neurological diseases, and whether pericytes can be targeted therapeutically to correct for neurovascular and neuronal dysfunction associated with these neurological disorders.

## Data Availability Statement

The datasets generated for this study are available on request to the corresponding author.

## Ethics Statement

The animal study was reviewed and approved by Institutional Animal Care and Use Committee at the University of Southern California.

## Author Contributions

AN planned and performed *in vivo* imaging experiments, data analysis and prepared the manuscript. MS assisted in data analyses. YW performed cranial window surgeries. KK developed protocols for capillary diameter and red blood cells (RBC) velocity analysis and discussed results. BZ and ZZ co-developed the pericyte-CreER mouse model. BZ planned experiments and wrote the manuscript.

## Conflict of Interest

The authors declare that the research was conducted in the absence of any commercial or financial relationships that could be construed as a potential conflict of interest.
