# Supplementary figures and images for: Channelrhodopsin Excitation Contracts Brain Pericytes and Reduces Blood Flow in the Aging Mouse Brain in vivo
Source: Front Aging Neurosci. 2020 Apr 29;12:108. doi: 10.3389/fnagi.2020.00108 (PMC7201096; doi:10.3389/fnagi.2020.00108)

## Slide 1
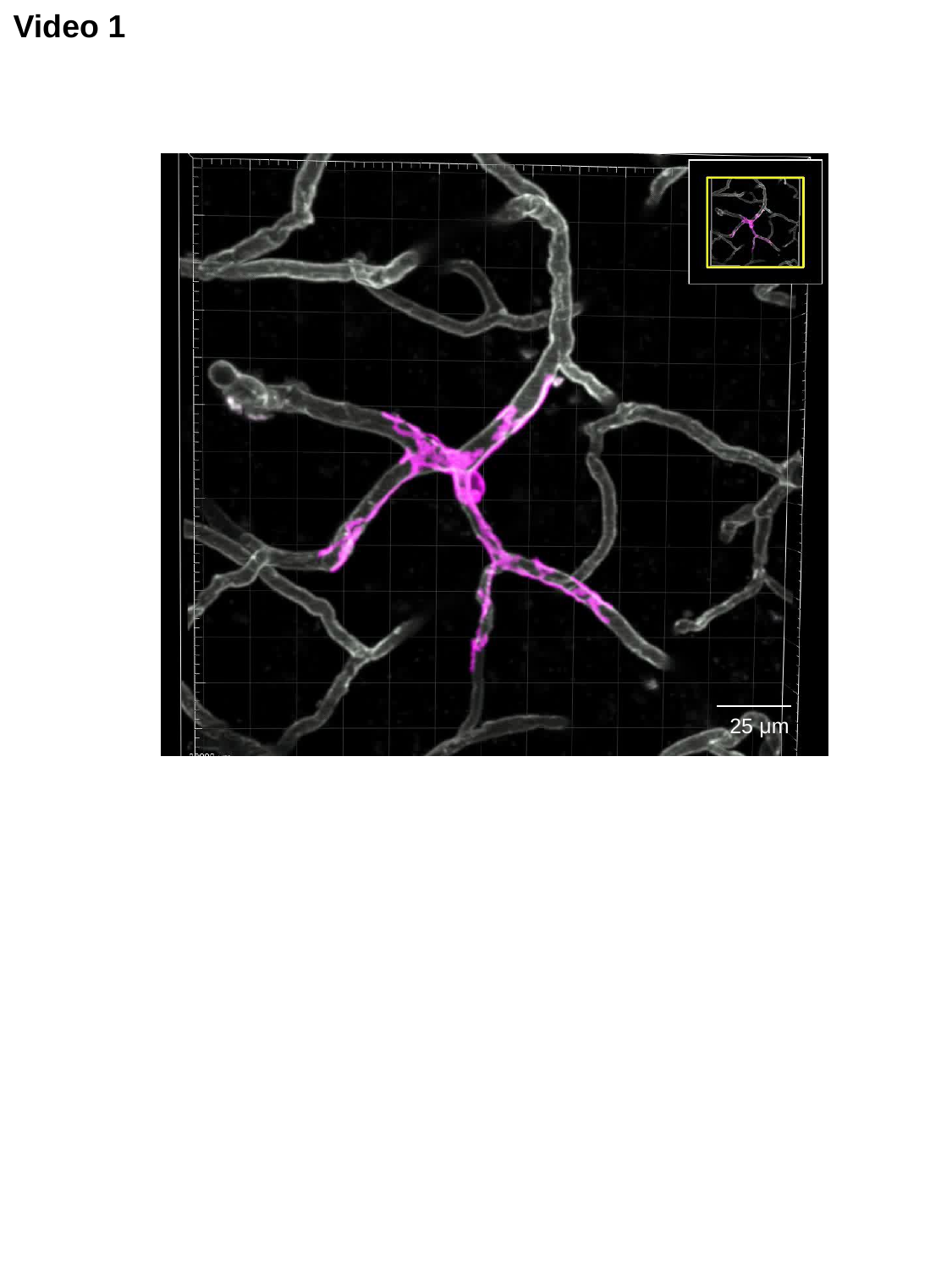

Video 1
25 μm

## Slide 2
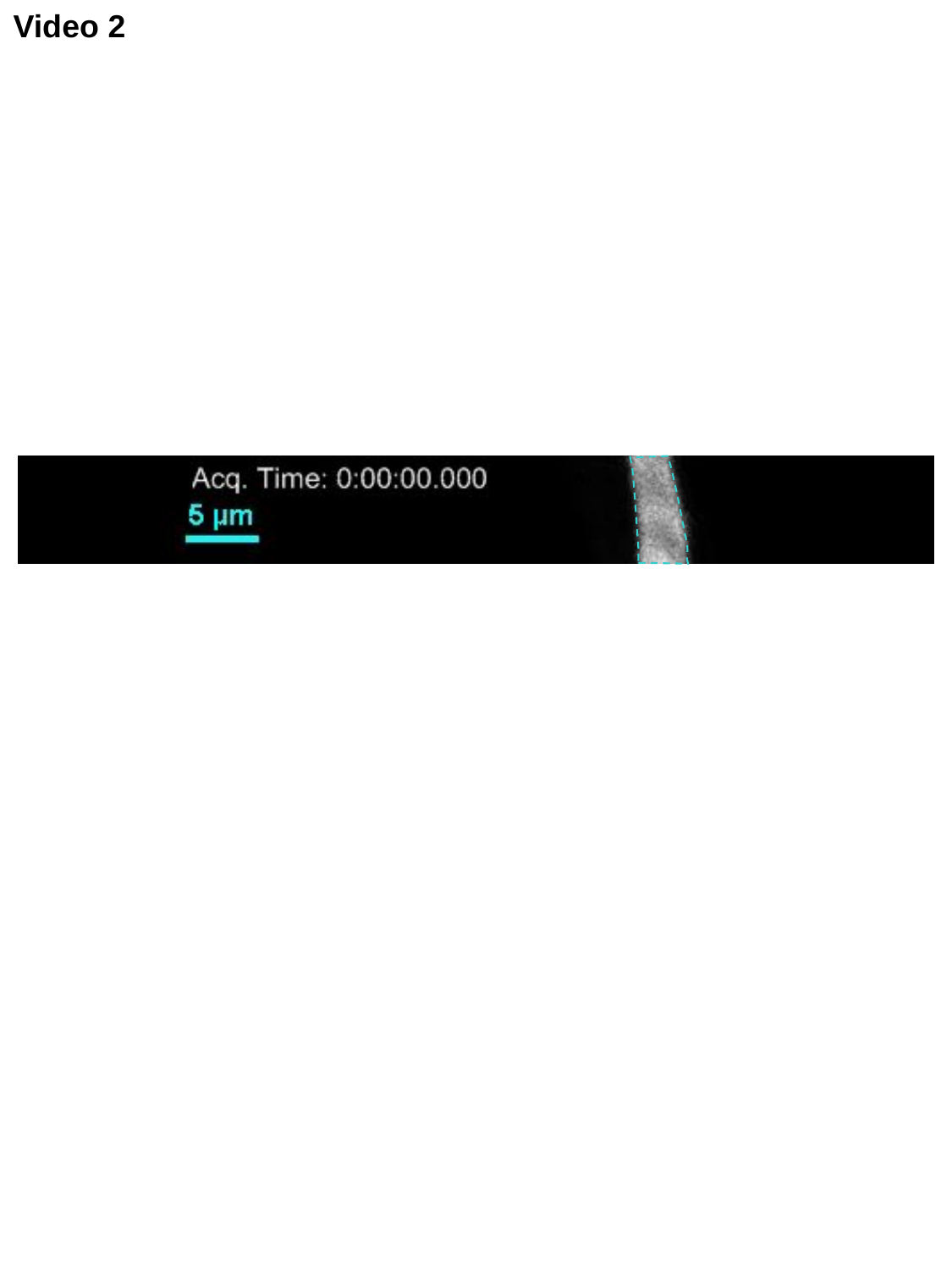

Video 2

## Slide 3
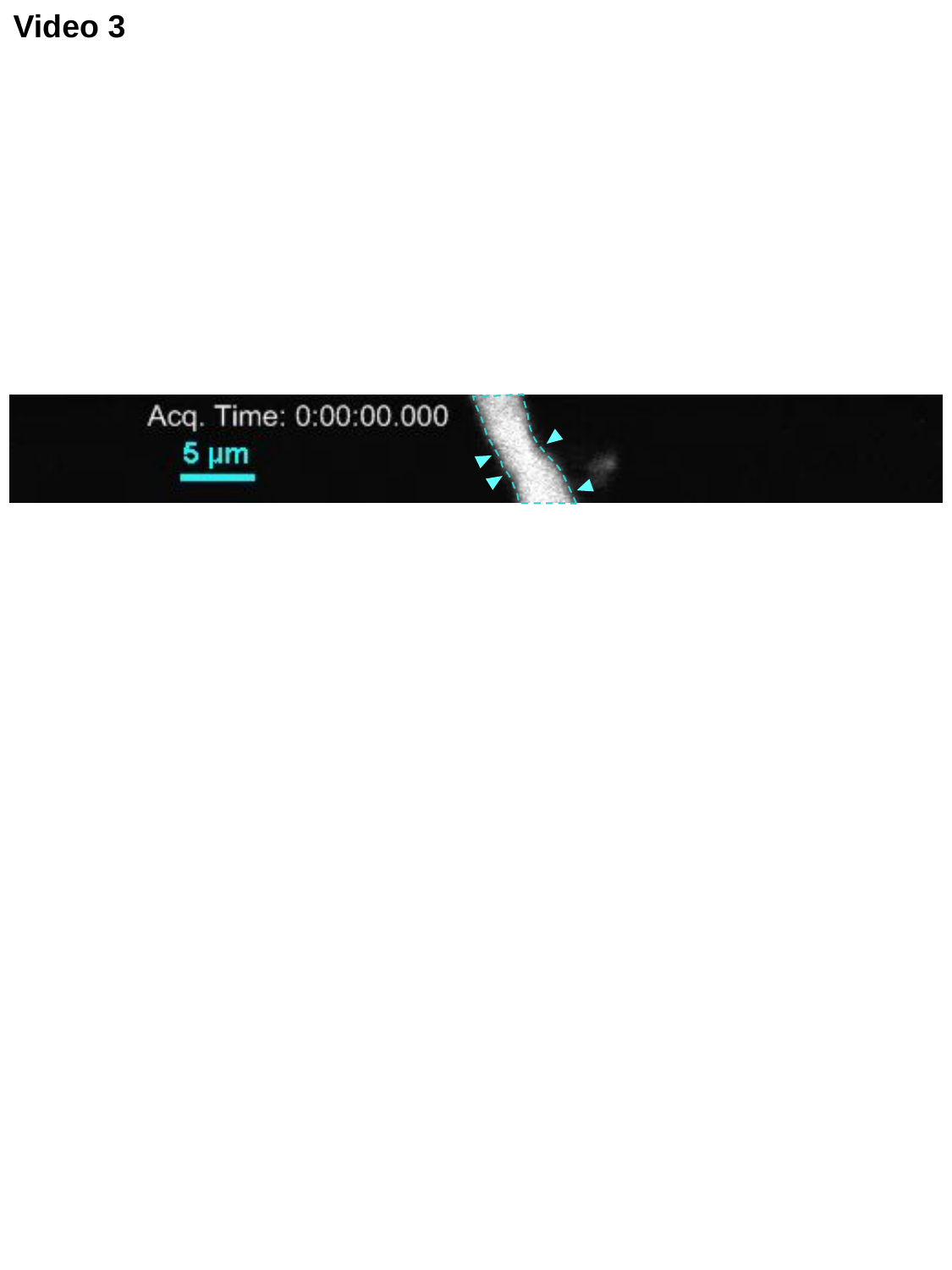

Video 3

Supplement: Supplementary file 1 [file Presentation_1.PPTX]
