# Supplementary material for: Channelrhodopsin Excitation Contracts Brain Pericytes and Reduces Blood Flow in the Aging Mouse Brain in vivo
Source: Front Aging Neurosci. 2020 Apr 29;12:108. doi: 10.3389/fnagi.2020.00108 (PMC7201096; doi:10.3389/fnagi.2020.00108)
Supplement: Supplementary file 2 [file Data_Sheet_1.docx]

Supplementary Material

# Supplementary Data

Please see below legends to the supplementary videos.

**Supplementary Video 1.** Video of Figure 1D showing three-dimensional visualization of a ChR2+ pericyte (fuchsia) along brain capillaries (gray).

**Supplementary Video 2.** Time-lapse video of region indicated in Figure 3A showing capillary flow of 70 kDa dextran and RBCs during stimulation of a ChR2- pericyte. Stimulation is indicated by a cyan dot in the upper right-hand corner. Cyan dashed line indicates pre-stimulation diameter.

**Supplementary Video 3.** Time-lapse video of region indicated in Figure 3C showing capillary flow of 70 kDa dextran and RBCs during stimulation of a ChR2+ pericyte. Stimulation is indicated by a cyan dot in the upper right-hand corner. Cyan dashed line indicates pre-stimulation diameter. Arrows indicate locations of pronounced vessel constriction. Dark shadows passing through the vessel are RBCs and are particularly apparent at later times in the movie due to reduced velocity at constriction.
